# Survey on sodium and potassium intake in patients with hypertension in China

**DOI:** 10.1111/jch.14355

**Published:** 2021-09-25

**Authors:** Ningling Sun, Yinong Jiang, Hongyi Wang, Yifang Yuan, Wenli Cheng, Qinghua Han, Hong Yuan, Li Yang, Zihong Guo, Yuemin Sun, Gang Sun, Xinhua Yin, Hao Wang, Jianjun Mu, Jiguang Wang

**Affiliations:** ^1^ Institute of hypertension People's Hospital, Peking University Beijing China; ^2^ The Institute of Hypertension and Heart Failure The 1st Affiliated Hospital of Dalian Medical University Dalian China; ^3^ Department of Cardiology Tsinghua Changgung Hospital Tsinghua University Beijing China; ^4^ Department of Hypertension Beijing Anzhen Hospital Affiliated to Capital Medical University Beijing China; ^5^ Department of Cardiology First Hospital of Shanxi Medical University Taiyuan China; ^6^ Department of Hypertension Third Xiangya Hospital Central South University Changsha China; ^7^ Department of Geriatrics Yan'an Affiliated Hospital of Kunming Medical University Kunming China; ^8^ Hypertension ward Fuwai Yunnan Cardiovascular Hospital Beijing China; ^9^ Department of Cardiology Tianjin Medical University General Hospital Tianjin China; ^10^ Department of Cardiology The Second Affiliated Hospital of Baotou Medical College Baotou China; ^11^ Department of Cardiology The First Affiliated Hospital of Harbin Medical University Harbin China; ^12^ Department of Hypertension Henan Provincial People's Hospital Zhengzhou China; ^13^ Department of cardiology First Hospital of Xi'an Jiaotong University Xi'an China; ^14^ Shanghai Institute of Hypertension Ruijin Hospital Shanghai Jiaotong University School of Medicine Shanghai China

**Keywords:** 24 h urinary sodium and potassium excretion, hypertension, survey, urinary sodium/potassium ratio

## Abstract

Sodium and potassium intake in hypertensive patients in China is not clear. The authors aimed to investigate the distribution of sodium and potassium intake in hypertensive patients in China, and to analyze the relationship between sodium and potassium intake and blood pressure. The study was performed in 130 hospitals from 23 provinces across China from 2016 to 2019. Finally, 9501 hypertensive patients average aged 54 years were included. 24 h urinary sodium and potassium excretion were measured. Distribution of urinary electrolytes were described according to age, gender and region. The association between urinary electrolytes and blood pressure was analyzed by multivariate linear regression. Hypertensive patients exhibited an average 24 h urinary sodium and potassium excretion of 156.7 ± 81.5 mmol/d and 39.2 ± 20.2 mmol/d (equivalent to sodium chloride of 9.2 g/d, potassium chloride of 2.9 g/d), sodium/potassium ratio (median) of 4.14 (2.92,5.73). Urinary electrolytes were lower in women than men (sodium: 171.1 vs 138.7, *p* < .05; potassium: 40.3 vs 37.7, *p* < .05), in the elderly than in the younger (sodium: 168.7 vs 139.9, *p* < .05; potassium: 39.5 vs. 37.5, *p* < .05). For every 1 unit of Na/K ratio increase, blood pressure increased by 0.46/0.24 mmHg. Blood pressure was 2.75/1.27 mmHg higher in quartile 4 than quartile 1 of Na/K. It remains high sodium and low potassium for hypertensive patients in China. Decreased sodium, Na/K ratio and increased potassium may help for blood pressure management.

## 1 INTRODUCTION

Both sodium and potassium are crucial nutrients; however, excess sodium intake and insufficient potassium intake increase the risk of hypertension.^1^, ^2^ The INTERSALT^3^ study found that increased 24 h urinary sodium excretion was associated with increased blood pressure. A balanced diet of sodium and potassium intake is essential for cardiovascular health. The WHO^4^ recommended that the daily sodium intake does not exceed 85 mmol/d, and that the daily potassium intake is at least 90 mmol/d. The “Chinese guidelines for the management of hypertension” (2018 revised edition) recommends that the daily salt intake does not exceed 100 mmol/d.^5^ Studies in 2012 demonstrated that the average sodium intake of Chinese residents was 10.5 g/d,^6^ which is equivalent to 180 mmol/day for urinary sodium excretion. However, there is a lack of data on salt intake of hypertensive patients in China, even though balance sodium and potassium pattern are important for blood pressure management in such patients. Given the pivotal role of salt intake in blood pressure management and its lack of epidemiological data on in hypertensive patients, we conducted a nationwide survey on sodium and potassium intake in hypertensive patients. The aim of our study was to: (1) describe the current epidemiology of urine electrolytes for hypertensive patients in China, especially by subgroups including age, gender, and region. (2) analyze the association between urinary electrolytes and blood pressure.

## 2 METHODS

This cross‐sectional study used data from the survey under China Health Action on Salt and Hypertension (CASH) program. This program aimed to reduce the average salt intake from 10.5 g/d to less than 5 g/d by 2030 in China. To evaluate sodium and potassium intake in hypertensive patients, 24 h urinary electrolytes were measured in patients with essential hypertension in 130 hospitals of 23 provinces in China between October 2016 and October 2019. This study has been approved by the ethics committee of Peking University People's Hospital, and informed consent was obtained (N0.2016PHB074‐01).

### 2.1 Survey design

Hospitals were recruited according to the geographical distribution, namely each province chooses one tertiary hospital (more than 1000 beds and has hypertension ward) as the leading unit, and each tertiary hospital organizes 5–10 subordinate hospitals to participate in the study. Only hospitals that were able to conduct 24 h urinary electrolyte measurements were included. The working team gathered all researchers in each region to introduce the investigation purpose, requirements and implementation plan of the investigation, to conduct training, and to provide SOPs before participants recruitment.

### 2.2 Study population

Participants were enrolled in the study according to the sequence of hospitalization in a consecutive manner. Patients’ informed consent were obtained before study. Inclusion criteria were as follows: (1) essential hypertensive patients according to 2010^7^ and 2018^5^ guidelines for the prevention and treatment of hypertension in China; (2) patients aged 18–85 years old. Hypertension was defined as follows: blood pressure was measured three times without the use of antihypertensive drugs, SBP was ≥140 mmHg (1 mmHg = 0.133 kPa) and DBP was ≥90 mmHg, or patients with hypertension history and currently on antihypertensive medication with blood pressure < 140/90 mmHg. All patients had a regular diet (three meals a day) for nearly a month.

The exclusion criteria were as follows. (1) Patients with serious systemic diseases (i.e., rheumatic immunity and tumors), secondary hypertension, and other conditions that could cause structural changes, remodeling of the heart, and serious complications (i.e., cardiomyopathy, valvular heart disease, contractile heart failure, pulmonary heart disease, and atrial fibrillation). (2) Patients with severe liver and kidney disease, infection, tumors, malnutrition, severe cardiorenal insufficiency, cardiocerebral vascular events in the past 6 months (myocardial infarction and stroke), hypertensive urgency, and poorly controlled diabetes (fasting bloodglucose > 11.1 mmol/L). (3) Patients using glucocorticoids. (4) Patients whose urine could not be collected all day or with a 24‐h urine output of < 500 ml or > 5000 ml.

### 2.3 Determination of 24 h urinary sodium and potassium

We provided 5‐liter urine buckets for each patient. Under a regular diet, 24 h excreted urine was collected, from 7:00 am on the first day and including the urine at 7:00 am on the second day, with the first urine sample discarded (Figure S1). If the urine retention time was < 24 h, the patient reported urine loss, and if the 24 h total urine volume was < 500 mL, the sample was excluded. The excretion of urinary electrolytes (sodium and potassium) throughout the day was expressed by mmol/d. The amount of excreted 24‐h urine sodium and potassium was calculated by multiplying the total volume of the collected urine by the concentration of sodium and potassium measured. Urine electrolyte was measured by ion selective electrode method with American Beckman's and Roche Diagnostics biochemical testing equipment in all hospitals involved in the survey. Each laboratory must accept the inter‐laboratory quality assessment of potassium and sodium organized by National Health Commission's clinical testing centre, and those which have passed the examination entered the study. We give standard sodium and potassium samples of low, medium and high concentration respectively to hospitals’ laboratory and the coefficient of variation between different hospitals must less than 10%. Pearson correlation coefficient of sodium was 0.999 and potassium 0.989, intra‐group correlation coefficient of sodium was 0.955 (95% CI 0.949‐0.999) and potassium 0.986 (95% CI 0.9800‐0.999).

### 2.4 Other covariates

Demographic variables: age, gender, habits of smoking and drinking, medication, medical history, height (cm), weight (kg), and body mass index (BMI) = weight/height (kg/m^2^) were recorded for all participants.

Methods of office blood pressure measurement: Blood pressure was measured on the day of admission according to standard protocol. Participants were quietly seated and did not smoke, drink coffee/tea, and emptied their bladder 30 min before blood pressure measurement. The upper arm was exposed and at heart level. Either mercury sphygmomanometer or OMRON automatic digital BP monitor (Omron HEM‐1300, OMRON Healthcare, ON, China) was used to measure blood pressure according to clinical practice in each centre. Most patients used adult standard cuff (size 12 × 24 cm). If the upper arm circumference was larger than 36 cm, large cuff was used (size 16 × 30 cm) and if the upper arm circumference was smaller than 27 cm, small cuff was used (size 12 × 22 cm). Two blood pressure readings were taken on the right arm at an interval of 1–2 min. The average of the two readings was calculated.

### 2.5 Statistical analysis

Statistical analysis was performed with SAS 9.4 software (Cary, NC).

We first compared gender differences for baseline characteristics. Continuous and categorical variables were reported as mean ± SD, median (IQR), and n (%) and tested by t‐test, Mann Whitney U test, and χ^2^ test where appropriate. The sodium/potassium ratio was reported by median (mmol/d) due to skewed distribution. One‐way analysis of variance was used for comparison between multiple groups and Tukey method was used for post‐hoc pairwise comparison. Study population was categorized as below: (1) by age: < 45, 45–65, ≥65 years old (2) by gender:men and women (3) by geographical distribution of seven regions and their provinces: northwest (Gansu, Shanxi, and Xinjiang Uygur autonomous region), northeast (Heilongjiang, Jilin, and Liaoning), southwest (Guizhou, Sichuan, Yunnan, and Chongqing), east (Shandong, Shanghai, Zhejiang, Anhui, and Jiangxi), central (Henan and Hunan), south (Guangdong), and north China (Beijing, Tianjin, Inner Mongolia autonomous region, Shanxi, and Hebei). To analyze the association between Na/K ratio and blood pressure, multivariate linear regression analysis was conducted, adjusted for age, gender, BMI, and use of hypertensive medication. *P* < .05 was considered statistically significant.

## 3 RESULTS

### 3.1 Baseline characteristics

**FIGURE 1:**
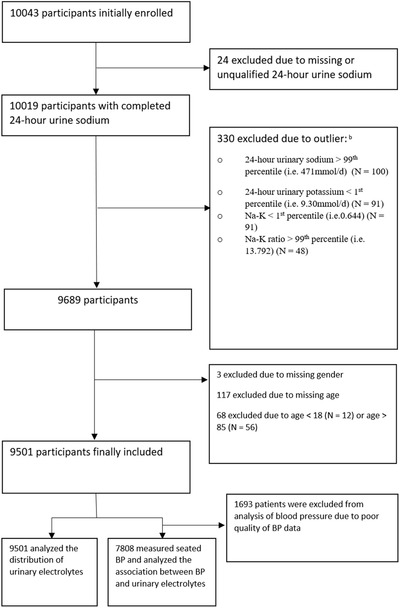
Screening procedures for patients with hypertension

A total of 9501 patients with hypertension from 130 hospitals in 23 provinces were finally included (Figure 1), with an average age of 56 ± 14.4 years, and women accounting for 44% (n = 4192). Among them, 7638 completed office blood pressure measurement. The mean systolic/diastolic blood pressure was 146/88 ± 21.2/15.2 mmHg, the mean heart rate was 76 ± 13.1 bpm, and BMI was 26 ± 3.8 kg/m^2^. A total of 81% of the participants received antihypertensive medication treatment (N = 6303). The percentage of antihypertensive medication use for ACEI, ARB, β‐blockers, CCB and low dose diuretics (Single‐Pill Combination included) was 10%, 24%, 19%, 64% and 9%, respectively. Urinary sodium was neither associated with antihypertensive medication use (*p* = .5645) nor diuretic use (*p* = .7838). The baseline characteristics of the patients are displayed in Table 1.

**TABLE 1:** Characteristics of the patients

Variable	All patients (N = 9501)	Men (N = 5309)	Women (N = 4192)	*P* value
Age (years)	55.73 ± 14.13	52.82 ± 14.38	59.40 ± 12.91	<.0001
Body mass index (kg/m^2^)	26.00 ± 3.83	26.50 ± 3.75	25.37 ± 3.83	<.0001
Systemic blood pressure (mmHg)	146.00 ± 21.18	145.94 ± 21.3	146.08 ± 21.03	.77
Diastolic blood pressure (mmHg)	87.78 ± 15.19	89.99 ± 15.51	85.00 ± 14.29	<.0001
Heart rate (bpm)	76.10 ± 13.16	77.05 ± 13.17	74.89 ± 13.04	<.0001
Sodium excretion (mmol/d)	156.69 ± 81.49	170.92 ± 85.17	138.67 ± 72.68	<.0001
Sodium chloride (g/d)	9.16 ± 4.77	10.00 ± 4.98	8.11 ± 4.25	<.0001
Potassium excretion (mmol/d)	39.15 ± 20.24	40.29 ± 20.98	37.70 ± 19.17	<.0001
Potassium chloride (g/d)	2.92 ± 1.51	3.00 ± 1.56	2.81 ± 1.43	<.0001
Sodium/Potassium Ratio	4.14 (2.92,5.73)	4.42 (3.16,6.17)	3.79 (2.67,5.24)	<.0001
Urine volume(ml)	1740 (1250,2290)	1750 (1300,2300)	1700 (1200,2200)	0.0250
Urinary creatine (umol/d)	9008 (5080, 12922)	11099 (6232, 14690)	7513 (4599,9747)	<.0001
Antihypertensive drug (%)	6303 (81.38%)	3514 (80.89 %)	2789 (82.01%)	.2121

*Note*: The data with normal distributions are expressed as (x±s). The median is used for data with non‐normal distributions (P25‐P75). Different scales of electrolytes were calculated as follows: sodium chloride (NaCl; g/d) = urinary sodium excretion (mmol/d) × 5.85 (g), and potassium chloride (KCl; g/d) = urinary potassium excretion (mmol/d)×7.45 (g).

### 3.2 Distribution of sodium, potassium, and sodium/potassium ratio in hypertensive patients

For all hypertensive patients, the mean 24‐h urinary sodium was 156.8 ± 81.50 mmol/d (i.e., 9.2 g/d of sodium chloride). The mean urinary potassium was 39 ± 20.24 mmol/d (i.e., 2.9 g/d of potassium chloride). The median sodium/potassium ratio was 4.14. The proportion of patients with low (< 100 mmol/d), medium (100‐200 mmol/d), and high (≥200 mmol/d)^3^ urinary sodium was 26.7%, 47.9%, and 25.4%, respectively (Figure S2).

### 3.3 Distribution of urinary sodium and potassium by age and gender

**FIGURE 2:**
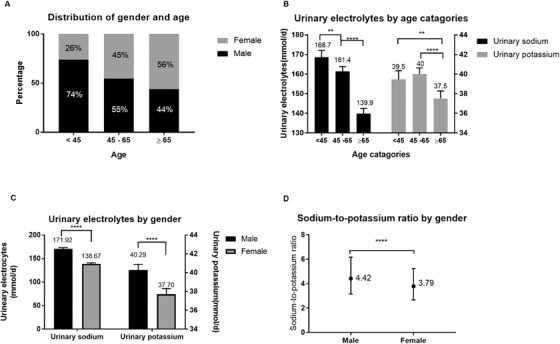
Age and gender distributions of patients (A). Urinary sodium and potassium according to age (B) and gender (C), Urinary sodium/potassium ratio according to gender (D). **P < .05, ****P < .0001

The age distribution was as follows: < 45 years old (n = 2170, 23%), 45–65 years old (n = 4514, 47%), and > 65 years old (n = 2817, 30%). Altogether there were 5309 men and 4192 women. The proportion of women was higher in the older age group (Figure 2A). The mean values of urinary sodium in the three groups were 169 ± 84.5 mmol/d, 161 ± 83.9 mmol/d, and 140 ± 72.1 mmol/d, respectively. Urinary sodium and potassium gradually decreased with increased age (Figure 2B). The urinary sodium, urinary potassium, and sodium/potassium ratio in women was lower than that in men (Figure 2C‐D).

### 3.4 Distribution of urinary sodium and potassium by urinary sodium excretion level

The average urinary sodium was 69.8 ± 21.0 mmol/d, 145.7 ± 28.0 mmol/d, and 268.8 ± 58.9 mmol/d in the low, medium, and high sodium groups, respectively. The average urinary potassium was 27.8 ± 13.1 mmol/d, 38.8 ± 17.8 mmol/d, and 49.7 ± 23.7 mmol/d, respectively (Figure S3A). High sodium intake (> 200 mmol/d) was significantly higher in men than women (Figure S3B). The sodium/potassium ratio was 2.72 (1.96,3.70), 4.09 (3.09,5.39), and 5.81 (4.48,7.46), respectively, and greater in patients with high sodium intake, indicating an increased ratio with increased sodium intake (Figure S3C).

### 3.5 Distribution of urinary sodium and potassium by region

A total of 130 hospitals in 23 provinces and cities were included in this study, and 24 h urinary sodium value of > 156.8 mmol/d (i.e., sodium chloride > 9.2 g/d) were in 14 provinces and cities. Salt intake was > 6 g/d in all 23 provinces and cities involved in the study (Figure S4A), whereas potassium intake was generally insufficient (Figure S4B). Highest salt excretion (184.5 ± 77.8 mmol/d) was found in northwest China, which is equivalent to salt intake 11.1 g/d (Figure S4C). Furthermore, the urinary sodium/potassium ratio in south China (2.8(1.9,3.7)) was significantly lower than that in other areas (Figure S4D).

### 3.6 Relationship between sodium/potassium ratio and office blood pressure

**FIGURE 3:**
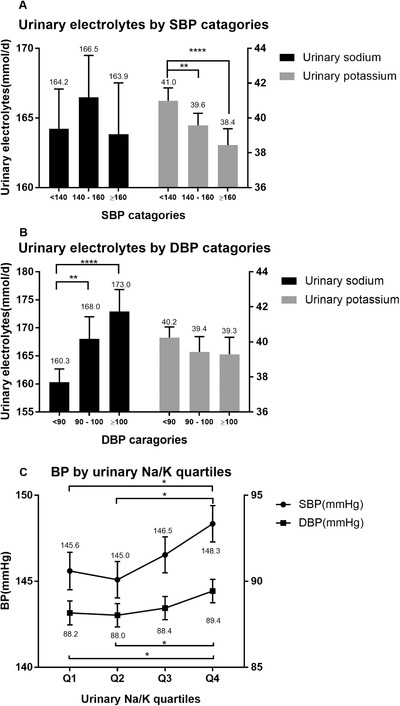
A) The sodium/potassium ratio under different systolic blood pressure (SBP) levels. B) The sodium/potassium ratio under different diastolic blood pressure (DBP) levels. C) The correlation of blood pressure (BP) and sodium/potassium ratio quartiles

Univariate analysis showed that urinary sodium excretion had a non‐linear trend with systolic blood pressure (Figure 3A) but a linear association with diastolic blood pressure (Figure 3B). However, for urinary potassium, both systolic and diastolic blood pressure gradually decreased with the increase of potassium (Figure 3A, Figure 3B). As for the association between Na/K ratio and blood pressure, participants in the highest Na/K quartile had the highest systolic and diastolic blood pressure (Figure 3C). Then, we used two methods to analyze the relationship between office blood pressure and urinary sodium/potassium ratio. In multiple linear regression analysis, we found that with 1 unit increase of Na/K, blood pressure increased 0.46/0.24 mmHg (Table 2). In categorical variable analysis, participants were divided according to sodium/potassium ratio quartile (Q1: < 2.93, Q2: 2.9–4.1, Q3: 4.7–5.8, Q4: > 5.8), office blood pressure increased with Na/K category after adjusting for age, gender, BMI, and anti hypertensive medication use. Blood pressure in Na/K Q4 increased by 2.75/1.27 mmHg compared with Q1 (Table 3 and Figure 3C).

**TABLE 2:** Office blood pressure and sodium/potassium ratio as continuous variable

	Model 1 (N = 7638)		Model 2 (N = 6950)	
	Βeta (95% CI)	*p*	Βeta (95% CI)	*p*
Systolic blood pressure (mmHg)	0.43 (0.22, 0.64)	<.0001	0.46 (0.24, 0.69)	<.0001
Diastolic blood pressure (mmHg)	0.59 (0.43, 0.74)	<.0001	0.24 (0.10, 0.38)	.0008

*Note*: Model 1 is the univariate model. Model 2 adjusted for age, gender, BMI, and history of antihypertensive medication (Blood pressure for every 1 unit increase in sodium to potassium ratio).

**TABLE 3:** Office blood pressure and quartiles of the sodium/potassium ratio

		Systolic blood pressure (mmHg)			Diastolic blood pressure (mmHg)		
		Beta (95% CI)	*P*#	*P* *	Beta (95% CI)	*P*#	*P* *
Sodium/potassium ratio	Q2	‐0.50 (‐1.92, 0.91)	.484	<.0001	‐0.14 (‐1.04, 0.77)	.768	.008
	Q3	0.94 (‐0.46, 2.34)	.188		0.28 (‐0.62, 1.17)	.546	
	Q4	2.75 (1.34, 4.15)	.0001		1.27 (0.37, 2.17)	.006	

*Note*: the model adjusted for age, gender, BMI, and antihypertensive medication. #: *P* values for different quartile levels compared to the reference group (Q1 is a reference group).

* *P* values of the relationship between whole quartile and blood pressure. Sodium/potassium ratio: Q1: < 2.91, Q2: 2.91‐4.14, Q3: 4.14‐5.72, Q4: > 5.72.

## 4 DISCUSSION

China is a country with a high incidence of hypertension and cerebral stroke.^8^ High sodium intake can significantly increase blood pressure and increase cerebrovascular risk in sensitive patients.^9^ As such, moderate reductions in sodium intake can effectively reduce blood pressure.^10^, ^11^ Excessive sodium intake, insufficient potassium intake, and a low sodium/potassium intake ratio are important risk factors for hypertension in China.^15^ The INTERSALT^3^ study found that every 100 mmol/d (i.e. 2.3 g/d) increase in 24 h urinary sodium excretion was associated with an average increase in systolic/diastolic blood pressure by 5–7/2‐4 mmHg. The survey also found that the average intake of cooking salt in Chinese residents aged ≥18 was 10.5 g/din 2012.^6^

Our study suggested an excessive sodium but insufficient potassium consumption and a consequent high sodium/potassium intake ratio in Chinese hypertensive patients. The average intake was 9.2 g/d, 2.9 g/d for sodium and potassium, respectively. The median Na‐K ratio was 4.14. The WHO and 2030 China Health Action Plan set the goal to reduce sodium intake to < 5 g/d.^14^ Our study revealed that sodium intake was > 6 g/d in 73% of hypertensive patients and < 5 g/d in only 19% of hypertensive patients (data not shown). On the other hand, only 2% achieved the recommended potassium level of above 90 mmol/d. Increased sodium could increase plasma volume and activate sympathetic activity, which increased peripheral vascular resistance, reduced arterial elasticity, and increased blood pressure.^18^ Salt restriction and supplementation of potassium can partially improve high blood pressure and contribute to clinical prognosis.^2^, ^12^, ^13^ Again, our analysis demonstrated the association between urinary electrolytes and blood pressure. All the above urges the need for appropriate salt restriction strategies for hypertensive patients. In the past 10 years, China has carried out numerous activities and projects on salt control and hypertension prevention,^15^ which are also emphasized in lifestyle interventions in hypertension guidelines. The growing awareness of the importance of salt restriction in hypertensive patients brought by health education has facilitated blood pressure management. It is inspiring to see the great reduction of salt consumption in the Chinese population from 10.5 g/d to 9.2 g/d. The effect of salt reduction intervention was also reported in a recent study in one province in China. The progress on salt restriction is due to the efforts of our government,^15^ associations, and salt control workers. However, there still remains a lot to be done for sodium restriction and potassium supplement.

Moreover, our study also showed that among hypertensive patients, sodium intake is 17% higher in those younger than 45 years compared with those older than 65 years. Additional analysis showed similar results after BMI was adjusted (data not shown), which suggested that the higher consumption of salt may not simply be due to higher consumption of food but higher sodium content instead. This warranted further attention. With the urbanization of the country, the dietary patterns and habits have undergone significant changes. Most young people do not eat at home; about 60% of them eating out for at least two meals a day (results of our other questionnaire investigation study, data on file). As the salt content of takeaway and fast food cannot be controlled, it is likely that young people eat a diet with higher sodium content. On the contrary, the elderly are more likely to maintain traditional family eating habits. At present, the supply of low‐sodium chloride in the market, the nation's popularization of education regarding salt intake, and controlling the amount of salt used for cooking have begun to show effects. Our results suggest that the salt control strategies introduced within families could spread to every corner of society.

According to the regional distribution of salt content in hypertensive patients in China, northwest China had the highest salt content with an increased sodium/potassium ratio, whereas the intake of sodium chloride was the lowest in south China with a urinary sodium/potassium ratio significantly lower than those in other regions. This further demonstrates the special salt diet pattern in China: ‘high in the north, low in the south’. High sodium and low potassium were associated with increased blood pressure. A recent study in a general Chinese population, which used 24 h‐recall as measurement of diet electrolytes, showed similar findings for regional differences between north and south China.^16^

The focus of this study: a large‐scale epidemiological survey involving 130 hospitals in 23 provinces of China was conducted to examine the salt intake status of Chinese hypertensive patients. Our study also assessed salt intake levels of hypertensive patients of different ages. All the participating hospitals performed the implementation plan according to the unified SOP. In this study, we used 24 h urinary sodium and potassium excretion as the gold standard for evaluating salt intake.

Limitations of this study: In consideration of the financial limitations and maneuverability of the study, most 24 h urine collections were performed only once. And not all of the participants tested for urinary creatinine. The results would be more accurate with multiple urine collections to account for day‐to‐day variation as well as urinary creatinine measurement to adjust for urine sample completion. Besides, information about season and temperature was not collected, which may affect urinary electrolytes excretion and will be considered in following study. In addition, this study did not include certain provinces and cities (i.e., Tibet and Qinghai), which should be further covered in the future.

## CONFLICTS OF INTEREST

The authors have no conflict of interest to declare.

## AUTHOR CONTRIBUTIONS

Ningling Sun proposed the concept and design of this paper, participated in the research and provided a large number of research data. Jiguang Wang supervised the conduct of the study and gave timely guidance. Yifang Yuan conducted data analysis and was part of the writing of Methods and Results, Ningling Sun wrote the Introduction, and Discussion and fully discussed the research data with the statisticians. Dr. Sun and Jiguang Wang reviewed and edited the manuscript and contributed to the discussion section. Other coauthors have suggested modifications to the manuscript. All the authors contributed to data collection and discussion of the paper.

## FUNDING

None.

## Supporting information




**Figure S1**. Urine retention time 24 h a day.

**Figure S2**. Distribution of sodium excretion

**Figure S3**. Urinary electrolytes (A), gender (B), and sodium/potassium ratio (C) according to urinary sodium categories.

**Figure S4**. A) The distribution of urinary sodium in 23 provinces and cities. B) The distribution of urinary potassium in 23 provinces and cities. C) Sodium and potassium in seven regions. D) The sodium/potassium ratio according to seven regions.
