# Supplementary figures and images for: Survey on sodium and potassium intake in patients with hypertension in China
Source: J Clin Hypertens (Greenwich). 2021 Sep 25;23(11):1957–64. doi: 10.1111/jch.14355 (PMC8630600; doi:10.1111/jch.14355)

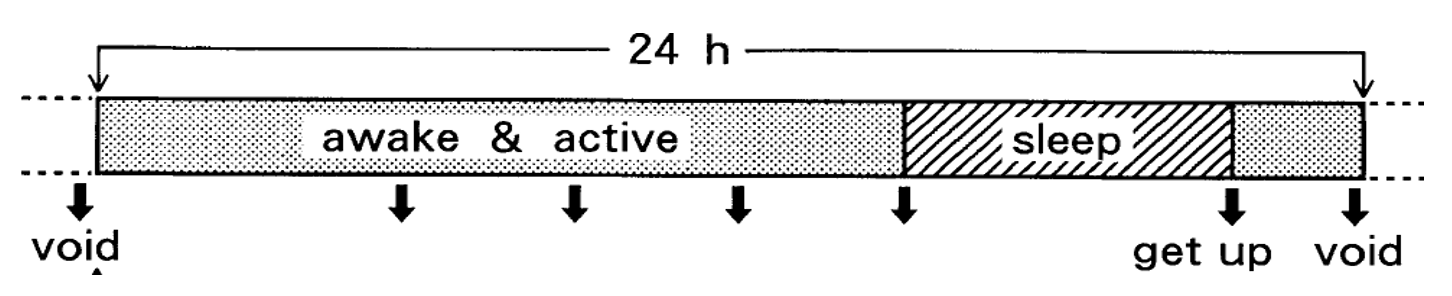

Supplement: Supplementary file 1 — Figure S1. Urine retention time 24 h a day. [file JCH-23-1957-s003.tif]

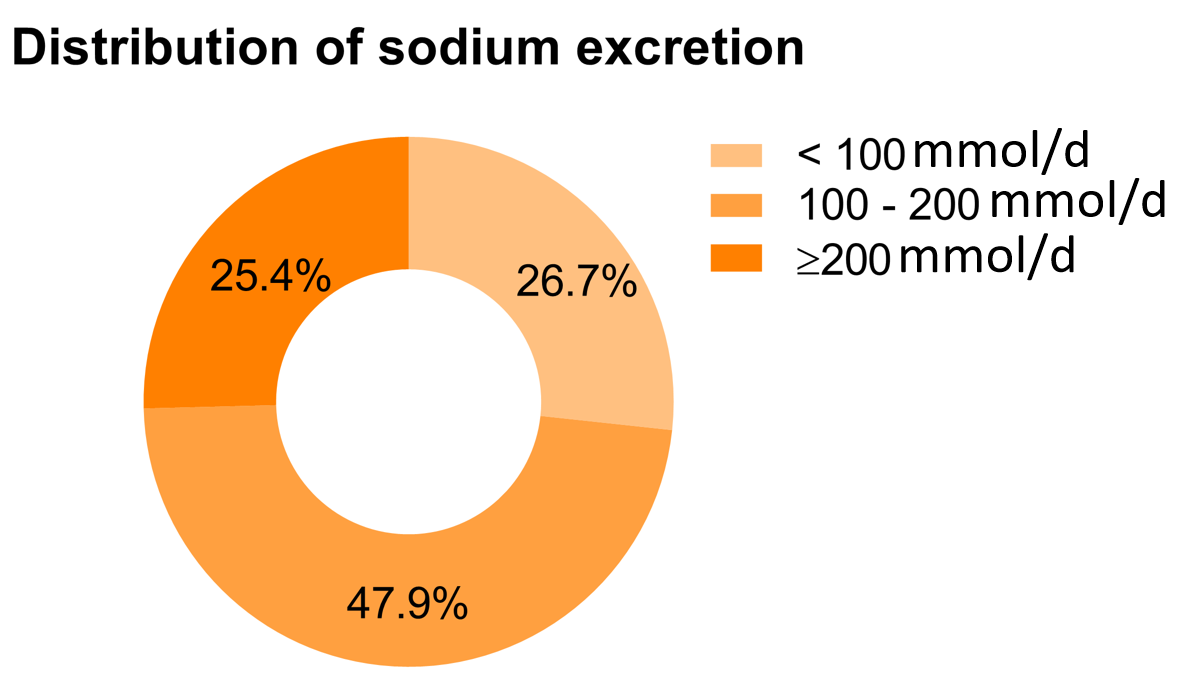

Supplement: Supplementary file 2 — Figure S2. Distribution of sodium excretion [file JCH-23-1957-s004.tif]

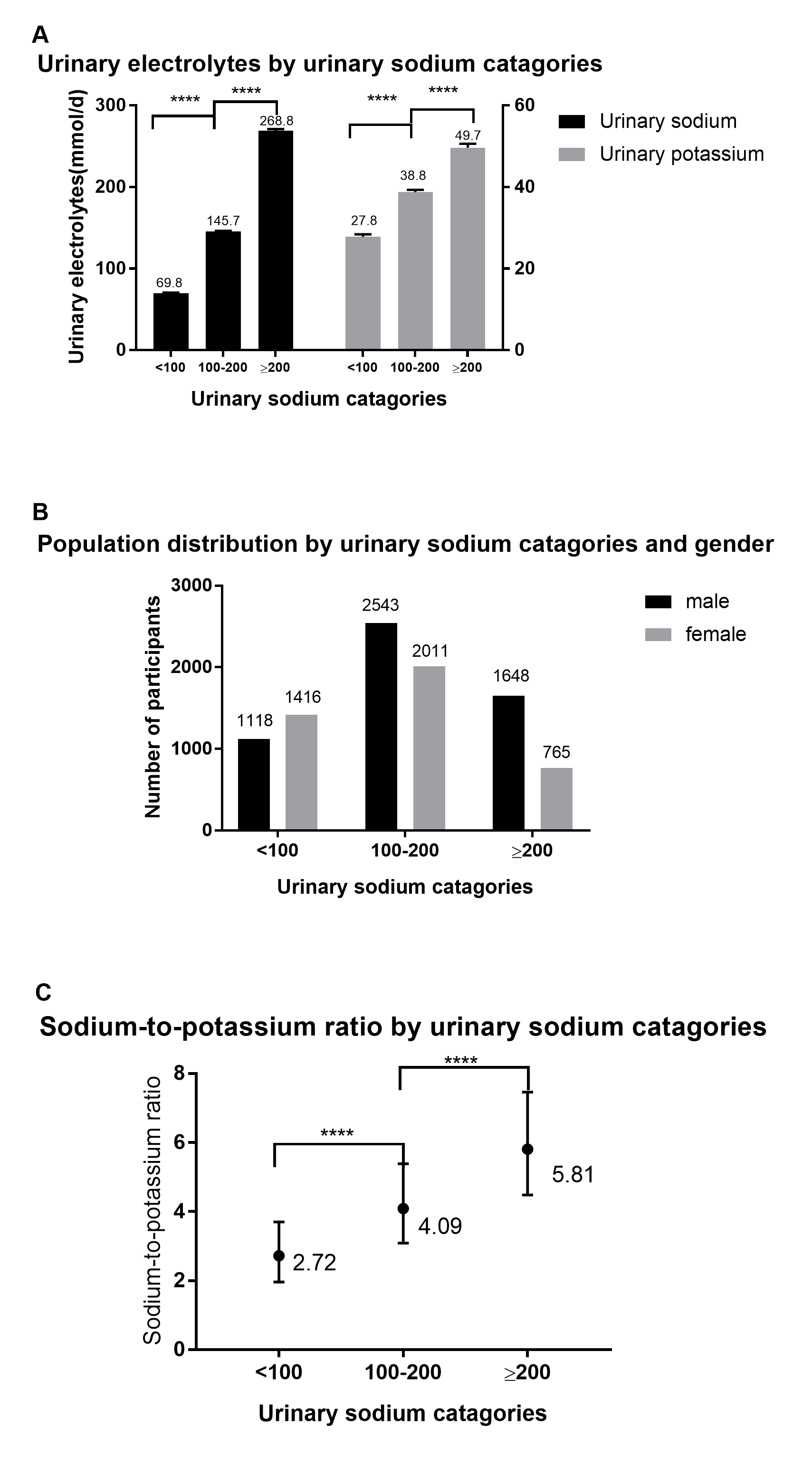

Supplement: Supplementary file 3 — Figure S3. Urinary electrolytes (A), gender (B), and sodium/potassium ratio (C) according to urinary sodium categories. [file JCH-23-1957-s001.tif]

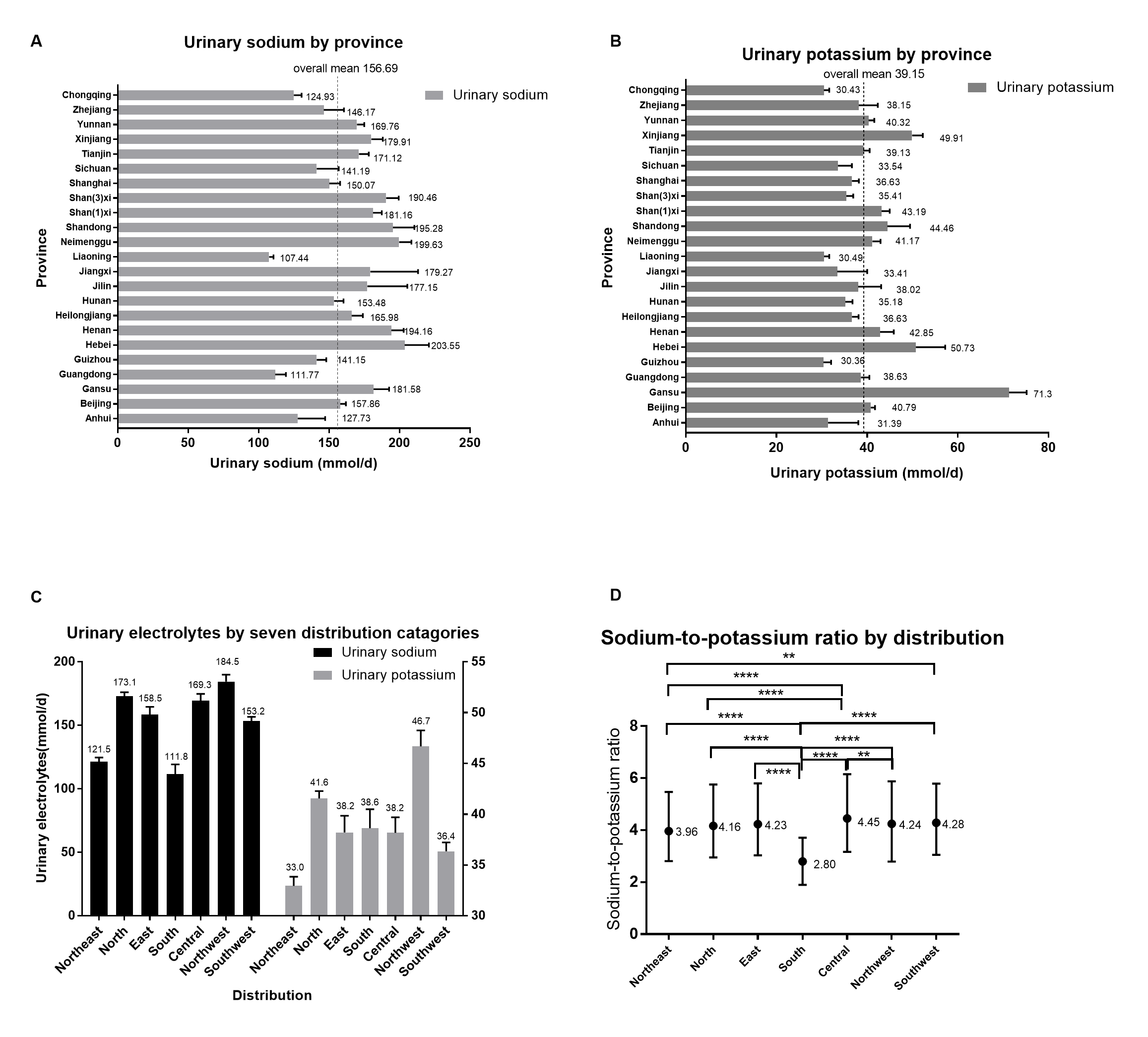

Supplement: Supplementary file 4 — Figure S4. A) The distribution of urinary sodium in 23 provinces and cities. B) The distribution of urinary potassium in 23 provinces and cities. C) Sodium and potassium in seven regions. D) The sodium/potassium ratio according to seven regions. [file JCH-23-1957-s002.tif]
